# Flammability and Thermal Kinetic Analysis of UiO-66-Based PMMA Polymer Composites

**DOI:** 10.3390/polym13234113

**Published:** 2021-11-26

**Authors:** Ruiqing Shen, Tian-Hao Yan, Rong Ma, Elizabeth Joseph, Yufeng Quan, Hong-Cai Zhou, Qingsheng Wang

**Affiliations:** 1 Artie McFerrin Department of Chemical Engineering, Texas A&M University, College Station, TX 77843, USA; ruiqing@tamu.edu (R.S.); marong1863@tamu.edu (R.M.); yquan@tamu.edu (Y.Q.); 2 Department of Chemistry, Texas A&M University, College Station, TX 77843, USA; thyan426@tamu.edu (T.-H.Y.); eajoseph@tamu.edu (E.J.); zhou@chem.tamu.edu (H.-C.Z.)

**Keywords:** metal–organic frameworks, polymer composite, pyrolysis kinetics, flame retardancy

## Abstract

Metal–organic frameworks (MOFs) are emerging as novel flame retardants for polymers, which, typically, can improve their thermal stability and flame retardancy. However, there is a lack of specific studies on the thermal decomposition kinetics of MOF-based polymer composites, although it is known that they are important for the modeling of flaming ignition, burning, and flame spread over them. The thermal decomposition mechanisms of poly (methyl methacrylate) (PMMA) have been well investigated, which makes PMMA an ideal polymer to evaluate how fillers affect its decomposition process and kinetics. Thus, in this study, UiO-66, a common type of MOF, was embedded into PMMA to form a composite. Based on the results from the microscale combustion calorimeter, the values of the apparent activation energy of PMMA/UiO-66 composites were calculated and compared against those of neat PMMA. Furthermore, under cone calorimeter tests, UiO-66, at only 1.5 wt%, can reduce the maximum burning intensity and average mass loss rate of PMMA by 14.3% and 12.4%, respectively. By combining UiO-66 and SiO_2_ to form a composite, it can contribute to forming a more compact protective layer, which shows a synergistic effect on reducing the maximum burning intensity and average mass loss rate of PMMA by 22.0% and 14.7%, respectively.

## 1. Introduction

Metal–organic frameworks (MOFs), also known as porous coordination polymers (PCPs), have emerged as a promising class of crystalline porous materials with unique properties [1]. They are essentially formed by connecting metal ions with polytopic organic linkers together through coordination bonds. Due to their exceptionally high specific surface area, tunable pore size distribution, and rich surface chemistry, MOFs have received significant interest in the areas of gas storage, gas/vapor separation, catalysis, luminescence, and drug delivery [2]. Most recently, they also have received much attention as a novel type of filler into polymers to form composites. Due to the inorganic–organic hybrid nature, MOFs usually have better compatibility with polymers to form polymer composites. These polymer composites also show promising flame retardancy and thermal stability [3,4,5]. However, despite the fact that knowing the thermal decomposition kinetics is important for the modeling of flaming ignition, burning, and flame spread over solid combustibles, there is a lack of studies on the complete thermal decomposition kinetics of MOF-based polymer composites.

Poly (methyl methacrylate) (PMMA) is a kind of widely used thermoplastic in the family of poly (acrylic ester)s. It is one of the polymers with the most widely studied thermal decomposition mechanisms. Due to its atypical decomposition, PMMA is the ideal clear model polymer matrix to study how other fillers may affect its decomposition process and kinetics [6,7,8]. UiO-66 is composed of Zr_6_O_4_(OH)_4_ nodes having six Zr^4+^ ions in octahedral geometry and four oxygen atoms or hydroxyl at the centers of each of the facets of the octahedra [9]. These nodes are coordinated with twelve terephthalate ligands in such a way that each Zr atom becomes coordinated with eight oxygen atoms in a square antiprismatic geometry. UiO-66 is characterized by remarkable thermal stability and high stability in a wide range of organic solvents and water, which makes UiO-66 able to withstand the processing conditions of various polymers without undergoing any significant degradations of its functions. Moreover, zirconium ions or compounds have been found to have certain flame-retardant effects because they can enhance or catalyze char formation through the dehydrogenation of the polymer [10]. These properties make UiO-66 a desirable candidate to be used as a flame-retardant filler. Furthermore, UiO-66 can form a composite with silica (SiO_2_) [11,12]. Silica has been proved to be an effective type of environmentally friendly flame-retardant filler for PMMA [13]. The SiO_2_@UiO-66 composite may combine the advantages of both components to further improve the thermal stability and flame retardancy of PMMA.

Therefore, in this study, UiO-66 was used as a novel type of flame-retardant filler for PMMA, in which how UiO-66 affects the thermal decomposition kinetics of PMMA was studied using a heat-release-based kinetic model. Furthermore, to improve the thermal stability and flame retardancy of PMMA, a SiO_2_@UiO-66 composite was also synthesized and then used as another type of flame-retardant filler. The structure and morphology of UiO-66 and its SiO_2_@UiO-66 composite were characterized by Fourier transform infrared spectroscopy (FTIR), powder X-ray diffraction (PXRD), and scanning electron microscopy (SEM). Their thermal stability and flame-retardant performance were evaluated by thermogravimetric analysis (TGA) and cone calorimeter tests.

## 2. Materials and Methods

### 2.1. Materials

All the reagents were obtained from commercial suppliers and used without further purification. Zirconium(IV) chloride (ZrCl_4_), terephthalic acid (H_2_BDC), acetic acid, concentrated hydrochloric acid (HCl), amorphous silicon dioxide (SiO_2_) (0.011 μm), and 2,2′-azobis(2-methylpropionitrile) (AIBN) were purchased from Sigma-Aldrich (St. Louis, MO, USA). N, N-dimethylformamide (DMF) was purchased from Fisher Scientific (Hampton, NH, USA). Methanol and methyl methacrylate (MMA) (99%, stabilized) were purchased from VWR (Radnor, PA, USA). Carboxylic-acid-functionalized silica (COOH SiO_2_) (20–30 nm) was purchased from US Research Nanomaterials, Inc. (Houston, TX, USA).

### 2.2. Synthesis of UiO-66

UiO-66 was prepared by a solvothermal method. First, ZrCl_4_ was dissolved in 10 mL of DMF, and 250 mg of H_2_BDC was dissolved in 20 mL of DMF with ultrasonication for 2 min. Then, these two solutions were mixed together. A 2 mL volume of concentrated HCl was added to the mixture and ultrasonicated for another 2 min. After this, the solution was kept in an oven with the temperature held at 120 °C for 72 h. After cooling down, the obtained white precipitate was centrifuged at 6000 rpm and washed thoroughly twice with DMF and another two times with methanol. Then, the synthesized UiO-66 was dried at 70 °C overnight until constant weight.

### 2.3. Synthesis of SiO_2_@UiO-66 Composite

**Figure 1:**
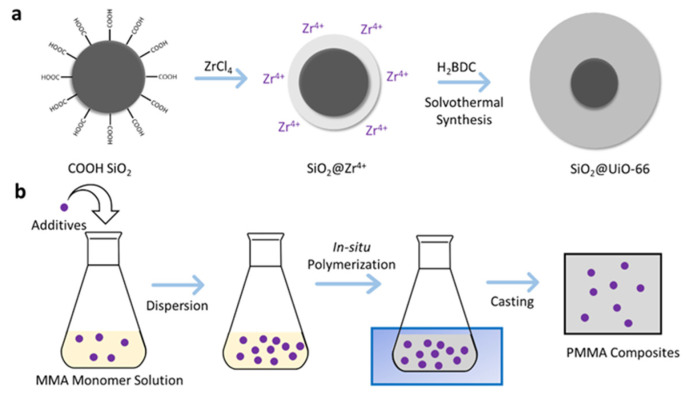
Synthesis of SiO_2_@UiO-66 composite (**a**); preparation of PMMA and its composites (**b**).

Synthesis of the SiO_2_@UiO-66 composite was carried out by immobilization of Zr^4+^ ions on COOH-functionalized SiO_2_ particles, followed by the solvothermal synthesis in the presence of H_2_BDC solution, as shown in Figure 1a. Briefly, ZrCl_4_ (0.64 g) was dissolved in 40 mL of DMF in a 250 mL round-bottom flask. COOH-functionalized SiO_2_ (0.5 g) was added and stirred at room temperature for 1 h to realize the immobilization of Zr^4+^ ions on the surface of SiO_2_ nanoparticles. Then, H_2_BDC (0.456 g) and acetic acid (4.0 mL) were dissolved in 40 mL of DMF, and then the solution was added to the mixture. The reaction was carried out at 120 °C under stirring and reflux condensing for 24 h. When cooling down, the prepared composites were collected by centrifugation at 1000 rpm and washed thoroughly by DMF, followed by drying under vacuum at 100 °C until constant weight.

### 2.4. Preparation of PMMA and Its Composites

PMMA and its composites were synthesized by in situ polymerization, as shown in Figure 1b. The monomer MMA was placed in a round-bottom flask with a silicone septum on top. Fillers were added to the monomer under continuous magnetic stirring. The stirring continued for half an hour, followed by sonication for another half an hour. The sonication procedure aided in the degasification of any dissolved oxygen. After sonication, the initiator AIBN was added at 0.2% of the total mass of MMA. To further remove dissolved oxygen, the solution was inerted by bubbling nitrogen gas through it for about 15 min. The solution was continuously stirred while the inert process took place. After the inerting process, the reaction vial was submerged in an oil bath maintained at a temperature of 70 °C with continuous magnetic stirring. Just before the solution gelling, the solution was poured into the curing mold, which is made of glass plates in parallel and silicone rubber between them to form a cavity with a thickness of 6 mm. After cooling down for one hour, the obtained solution was kept in a drying oven at 45 °C for 24 h to complete the curing process. Neat PMMA was prepared as a reference sample under the same conditions, just without adding any fillers.

### 2.5. Characterization and Measurements

Powder X-ray diffraction (PXRD) patterns of all the samples were obtained by using a Miniflex II (Rigaku, Tokyo, Japan) with Cu-Kα radiation (λ = 1.5406 Å), and the scanning range was 2θ from 5 to 60°.

Fourier transform infrared (FTIR) spectra were collected with a Nicolet iS5 spectrophotometer (Thermo Fisher Scientific, Waltham, MA, USA) equipped with iD7 ATR.

To study their surface morphological characterization, scanning electron microscopy (SEM) images of all samples were taken using JSM-7500F (JEOL, Tokyo, Japan). The samples were coated with platinum using a vacuum sputter coater first and then observed at 15 keV acceleration voltage. Composition analysis was performed on an energy-dispersive X-ray spectrometry (EDS) (Oxford EDS system, Abingdon, UK) with an acceleration voltage of 20 keV.

Thermogravimetric analysis (TGA) was carried out on a Q500 thermoanalyzer instrument (TA Instruments, New Castle, DE, USA). Approximately 8 mg of sample was heated from 30 °C to 600 °C at a heating rating of 20 °C/min, and the nitrogen atmosphere was maintained at a constant flow rate of 60.0 mL/min.

The cone calorimeter is a bench-scale instrument for testing the reaction-to-fire properties of materials under a forced-combustion condition [14,15]. In this study, cone calorimeter combustion tests were conducted on an iCone classic Calorimeter (Fire Testing Technology, West Sussex, UK) in accordance with the ASTM E-1354 standard operating procedure. All samples were with dimensions of 100 mm × 100 mm × 6 mm, and they were tested under an irradiance heat flux of 50 kW/m^2^ to simulate the ignition burner heat fluxes in the ASTM 84 or UL723 test [16]. A spark ignitor was also applied to ignite the pyrolysis gases.

The microscale combustion calorimeter (MCC) is pyrolysis-combustion flow calorimetry. It is a tool that utilizes milligram-sized samples to measure their pyrolysis and flammability behaviors when subjected to controlled heating in an atmosphere of nitrogen or synthetic air [17,18]. Moreover, MCC can also be used to derive the apparent kinetic parameters of the thermal decomposition of polymers based on a single-step reaction model. In this study, to simulate a real-fire scenario where the solid fuel undergoes anaerobic decomposition (as most of the oxygen is consumed in the flame zone), the sample, approximately 5 mg, was heated in a stream of nitrogen flowing at the rate of 80 cm^3^/min, and the thermal degradation products (fuel gases) were mixed with a 20 cm^3^/min stream of oxygen before entering a 900 °C combustion furnace. To derive the kinetic parameters, four heating rates of 0.25, 0.5, 1, and 1.5 K/s were considered. All measurements were performed using an MCC manufactured by Fire Testing Technology (West Sussex, UK), according to ASTM D7309 Method A.

## 3. Results and Discussion

### 3.1. Characterization of PMMA Additives

**Figure 2:**
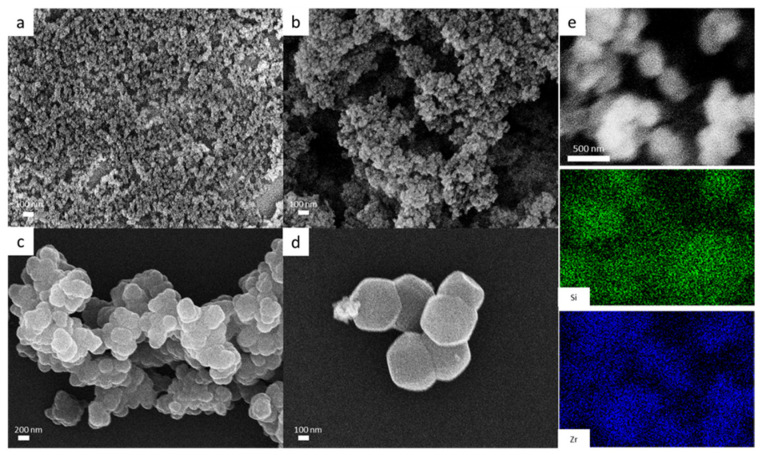
SEM images of (**a**) amorphous SiO_2_ nanoparticles, (**b**) COOH-functionalized SiO_2_, (**c**) UiO-66, and (**d**) SiO_2_@UiO-66 composite; (**e**) EDS spectrum of SiO_2_@UiO-66.

The COOH-functionalized silica nanoparticles were employed as nucleation cores for the growth and deposition of UiO-66 [19]. The COOH groups could bind Zr^4+^ to form the Zr^4+^@SiO_2_ precursor. Then, UiO-66 nanoparticles grew from the Zr^4+^@SiO_2_ precursor by the solvothermal synthesis in the presence of H_2_BDC ligand solution. The surface morphologies of amorphous SiO_2_ nanoparticles, COOH-functionalized SiO_2_, UiO-66, and the SiO_2_@UiO-66 composite are shown in Figure 2. From Figure 2a,b, it can be observed that both amorphous SiO_2_ and COOH-functionalized SiO_2_ have very small particle sizes, and they tend to aggregate together. Figure 2c,d demonstrates that the as-synthesized SiO_2_@UiO-66 composite has the same crystal shape as that of UiO-66, but its particle size is slightly larger than UiO-66 because of SiO_2_ inside the structure. The strong Si and Zr element signals from the EDX elemental mapping (Figure 2e) also indicate the successful growth and deposition of UiO-66 onto the Zr^4+^@SiO_2_ precursor.

**Figure 3:**
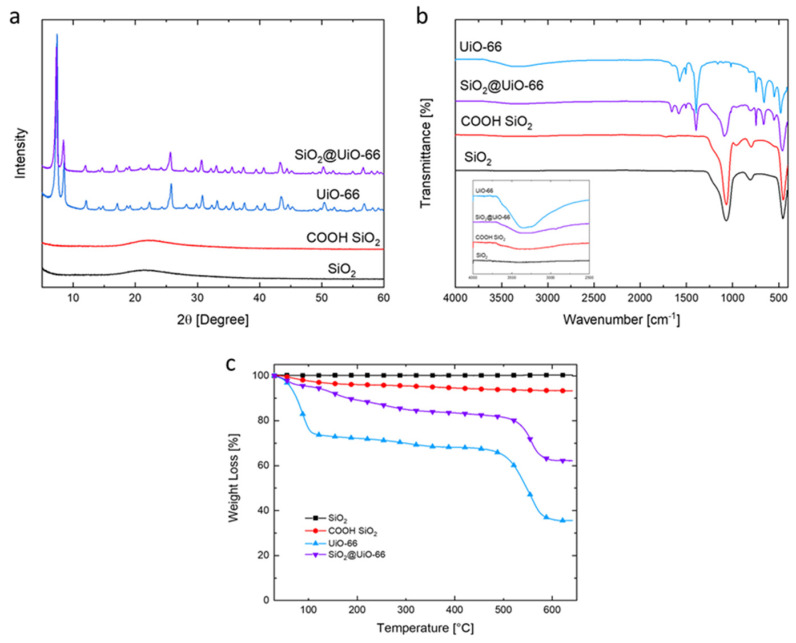
PXRD patterns (**a**), FTIR spectra (**b**), and TGA curves with respect to temperature in the nitrogen atmosphere (**c**) of amorphous SiO_2_ nanoparticles, COOH-functionalized SiO_2_, UiO-66, and SiO_2_@UiO-66 composite.

PXRD patterns of amorphous SiO_2_, COOH-functionalized SiO_2_, UiO-66, and the SiO_2_@UiO-66 composite are shown in Figure 3a. The PXRD patterns of amorphous SiO_2_ and COOH-functionalized SiO_2_ display a broad peak centered at 2θ = 23°, indicating their amorphous states. The characteristic diffraction peaks of UiO-66 are sharp and clearly identifiable, which shows a crystalline compound was obtained. The characteristic diffraction peaks at 2θ =  7.4°, 8.5°, and 25.7° match well with that previously reported and confirm the successful synthesis of UiO-66 [20]. For the SiO_2_@UiO-66 composite, it shows both the characteristic peaks of UiO-66 and the characteristic signal of COOH-functionalized SiO_2_ at 20–25°, which indicates that COOH-functionalized SiO_2_ does not noticeably influence the crystallization of the UiO-66 structures. However, the signal intensity of the characteristic peaks is observed to be lower than that of UiO-66, which is ascribed to the coordinate bonding of Zr with COOH of COOH-functionalized silica [21].

FTIR spectra were also recorded to confirm the chemical structure of amorphous SiO_2_, COOH-functionalized SiO_2_, UiO-66, and the SiO_2_@UiO-66 composite. As shown in Figure 3b, the FTIR spectrum of the amorphous SiO_2_ exhibits intense bands at 1068 and 794 cm^−1^, which correspond to the stretching vibration of Si–O–Si and the bending vibration of Si–O, respectively [22]. The appearance of –COOH characteristic bands at 3500–2500 cm^−1^ demonstrates the successful carboxyl modification of silica spheres [23]. The peak at 1717 cm^−1^ is related to the stretching vibration absorbance of C=O of the carboxyl groups [24]. In the FTIR spectra of UiO-66, the peak at 1656 cm^−1^ is assigned to the stretching vibrations of C=O in the carboxylic acid present in H_2_BDC, which indicates coordinate bonding of the metal with the organic fraction of terephthalic acid [21]. The peak at 1572 cm^−1^ is assigned to the O–C–O asymmetric stretching in the H_2_BDC ligand, the peak at 1507 cm^−1^ is assigned as the skeleton vibration of the benzene ring, and the peaks at 745 cm^−1^ indicate the para-substituent on the benzene ring [25]. For the SiO_2_@UiO-66 composite, all these characteristic peaks of UiO-66 can be identified, and new peaks appear at around 1087 cm^−1^ and 800 cm^−1^, which correspond to the stretching vibration of Si–O–Si and the bending vibration of Si–O groups of the silica core, respectively. Peaks at 1394 cm^−1^ confirm the bond between Zr^4+^ of UiO-66 and carboxylate-terminated silica [19].

Thermogravimetric analysis (TGA) results of those samples in the nitrogen atmosphere are shown in Figure 3c. For amorphous SiO_2_, its weight is quite constant during the heating in this temperature range, and no significant mass loss or gain process is observed. For COOH-functionalized SiO_2_, one main weight loss process is observed during the heating from the room temperature to 200 °C, which is mainly ascribed to the thermal decomposition of the COOH functional group attached to the SiO_2_ surface. For UiO-66, just as reported in the literature, a three-stage weight loss process is observed: the first stage (<100 °C) is related to the release of physisorbed water and the residual solvent trapped inside the porous structure of UiO-66; the second stage (100–450 °C) is related to the slow removal of dimethylformamide (DMF) and the dehydroxylation of the zirconium oxo-clusters; the third stage starts at 450 °C, which is also the major weight loss process, corresponding to the gradual decomposition of the organic ligand and the framework of UiO-66 [26]. At 600 °C, the final residue is 36.0 wt% of the initial mass of UiO-66. For the SiO_2_@UiO-66 composite, its weight loss process accounts for both the weight loss process of UiO-66 and the removal of organic groups of COOH-functionalized SiO_2_. Generally, it displays a similar shape of weight loss curve as that of UiO-66, but its final weight of residue at 600 °C (62.4 wt%) is much higher than that of UiO-66. The composition of the final residue is considered to be a mixture of SiO_2_ and ZrO_2_ [25]. Because of the presence of the thermally stable SiO_2_, it contributes to the higher amount of mass residue of the SiO_2_@UiO-66 composite.

### 3.2. Thermal Properties of PMMA and Its Composites

**Figure 4:**
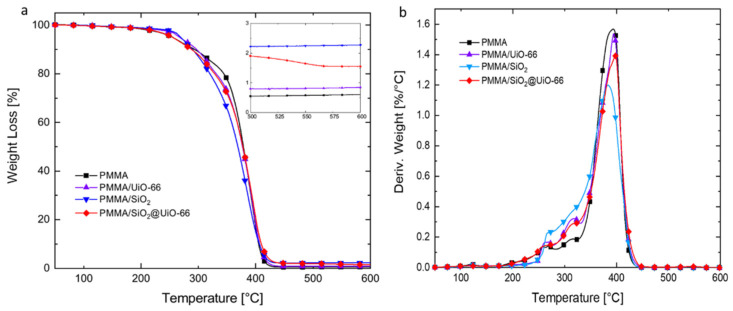
TGA (**a**) and DTG (**b**) curves of neat PMMA, PMMA/UiO-66, PMMA/SiO_2_, and PMMA/SiO_2_@UiO-66 with respect to temperature in nitrogen atmosphere.

In light of earlier research [27], in order to minimize the possible negative effects on the transparency and mechanical properties of PMMA, the mass loading of flame-retardant additives was kept at a low concentration of 1.5 wt%. The thermal stability of PMMA and its composites was evaluated using TGA under a nitrogen atmosphere. Their TGA and DTG (derivative thermogravimetric) curves with respect to temperature are shown in Figure 4a,b, respectively. The related data are listed in Table 1. As shown in Figure 4b, a four-stage thermal decomposition process was observed for PMMA. The first DTG peak is observed around 150 °C but is very small and negligible, which would correspond to the degradation step initiated by radical transfer to the unsaturated chain end. The second DTG peak (around 230 °C) and the third (around 270 °C) would be the result of the homolytic scission of the chain due to head-to-head linkages (H–H bonds) and of degradation initiated by radical transfer to unsaturated ends. Lastly, the fourth peak, which is also the main peak, would correspond to degradation initiated by random scission of the PMMA backbone [28].

With the addition of UiO-66 and its SiO_2_@UiO-66 composite, the general TGA and DTG curve shapes of PMMA remained the same, and the four-stage thermal decomposition process can still be clearly observed. As a comparison, with the addition of amorphous SiO_2_ only, the overlap between the second stage and the third stage is more pronounced. The initial thermal decomposition temperature (T_onset_) is defined as the temperature at which the sample has a weight loss of 10 wt%, and the maximum decomposition temperature (T_max_) is defined as the temperature at which the thermal decomposition rate of the sample reaches its maximum [29]. As presented in Table 1, the T_onset_ and T_max_ of PMMA are 293 and 394 °C, respectively, and its peak mass loss rate is 1.57 wt%/°C. With the addition of UiO-66, the T_onset_ and T_max_ of PMMA increase slightly to 297 and 395 °C, and the peak mass loss rate is reduced slightly to 1.54 wt%/°C. At this T_max_ (395 °C), the framework of UiO-66 still remains, and it can accumulate on the surface of the polymer melt, thus providing the mass and heat transfer barrier to slow down the mass loss process of PMMA. However, given their very close thermal decomposition behaviors, the addition of UiO-66 will not significantly change the thermal decomposition mechanisms of PMMA. As a comparison, with the addition of amorphous SiO_2_, although the T_onset_ and T_max_ of PMMA decrease slightly to 290 and 383 °C, its maximum mass loss rate (1.20 wt%/°C) is surprisingly lower than any other samples, which is ascribed to the “trapping effect” of SiO_2_ particles on the degradation products. The “trapping effect” of SiO_2_ particles also leads to the PMMA/SiO_2_ composite to reserve the maximum amount of mass (33.6 wt%) at the T_max_ [30]. For SiO_2_@UiO-66, its T_max_ (397 °C) is the highest among all samples. Taking advantage of the “trapping effect” of SiO_2_ particles and barrier effect of UiO-66, PMMA/SiO_2_@UiO-66 has a lower peak mass loss rate than PMMA/UiO-66. At 600 °C, the final residue of PMMA/SiO_2_@UiO-66 (1.6 wt%) is also higher than that of PMMA/UiO-66 (0.8 wt%), due to the presence of thermally stable SiO_2_ particles.

### 3.3. Flammability of PMMA and Its Composites

**Figure 5:**
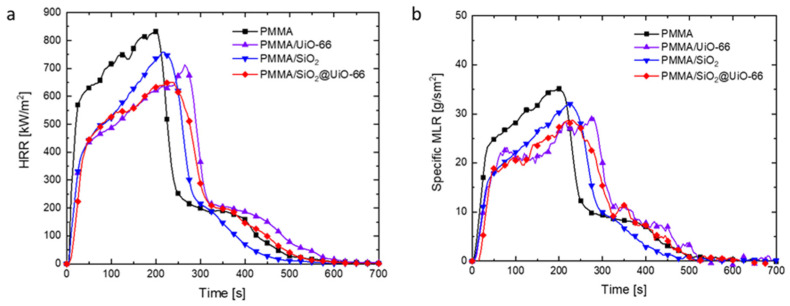
HRR (**a**) and specific MLR (**b**) curves of neat PMMA, PMMA/UiO-66, PMMA/SiO_2_, and PMMA/SiO_2_@UiO-66 with respect to time under cone calorimeter tests.

**Figure 6:**
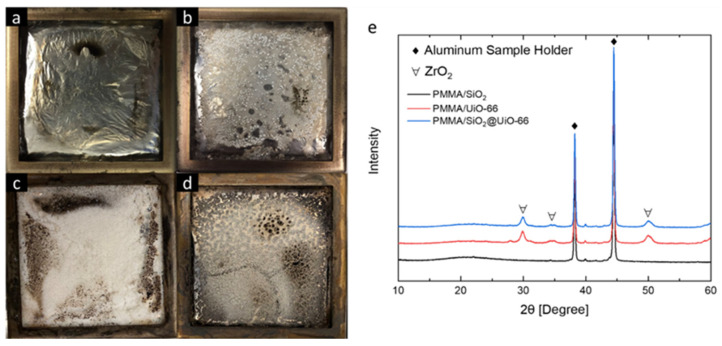
Combustion residues of neat PMMA (**a**), PMMA/UiO-66 (**b**), PMMA/SiO_2_ (**c**), and PMMA/SiO_2_@UiO-66 (**d**) after cone calorimeter tests; (**e**) PXRD patterns of combustion residues.

The reaction-to-fire properties of PMMA and its composites were comprehensively evaluated using the cone calorimeter, including their time to ignition, heat release rate (HRR), specific mass loss rate (specific MLR), combustion gas emission, effective heat of combustion (EHC), and fire load. The test results are summarized in Table 2. Figure 5a,b shows their HRR and specific MLR curves with respect to test time. Heat release and heat-driven mass loss are two important factors to assess fire hazards. From the HRR curve, both 6 mm thick PMMA and its composites show the burning behaviors of intermediate thick non-charring materials, in which HRR increases sharply after the ignition, but before reaching the peak value, the HRR increases at a slower rate [31]. Compared with neat PMMA, with only 1.5 wt% additives, the HRR of PMMA composites increases at a lower rate, and their final peak heat release rate (pHRR) is also lower. The pHRR of neat PMMA is 832 kW/m^2^. As a comparison, the pHRR of PMMA/UiO-66, PMMA/SiO_2_, and PMMA/SiO2@UiO-66 is 713, 758, and 649 kW/m^2^, which is reduced by 14.3%, 8.9%, and 22.0%, respectively. Besides pHRR, MARHE and average specific MLR are two additional parameters to evaluate the flammability of materials. The average rate of heat emission can be defined as the cumulative heat emission per unit time, and the peak value is considered as the maximum average rate of heat emission (MAHRE). MAHRE is a good parameter that can measure the tendency of the fire spread during a fire [32]. Average specific MLR is the average specimen mass loss rate per unit area (g/m^2^·s) computed over the period starting when 10 percent of the specimen mass loss occurred and ending when 90 percent of the specimen mass loss occurred, which better represents the mass loss process during the steady burning process. Fumed silica has shown to be an effective flame-retardant additive to reduce the burning intensity of PMMA by accumulating near the burning surface of the polymer and acting as a heat insulation shield to protect the polymer from further thermal decomposition [13]. In this study, PMMA/SiO_2_ also shows the same behavior. As shown in Figure 6c, after the cone calorimeter test, a white layer was left for PMMA/SiO_2_, which can act as a heat barrier and contribute to lowering the pHRR and MARHE during the flaming combustion. However, this layer is very loose, in which the pyrolysis products can transfer through it easily so that it cannot act effectively as a mass barrier. As shown in Figure 5b, although the peak specific MLR of PMMA/SiO_2_ is lower than that of PMMA, its average specific MLR is 24.0 g/sm^2^, which is even slightly higher than that of neat PMMA, around 22.5 g/sm^2^. Both PMMA/UiO-66 and PMMA/SiO_2_@UiO-66 show a better effect on slowing down the mass loss process and reducing the burning intensity of PMMA. Compared with PMMA/SiO_2_, both the mean HRR and average specific MLR of PMMA/UiO-66 are even lower. PMMA/UiO-66 has a mean HRR of 283 kW/m^2^ and an average specific MLR of 19.7 g/sm^2^. Due to the synergistic effect between SiO_2_ and UiO-66, PMMA/SiO_2_@UiO-66 has even lower values of mean HRR at 277 kW/m^2^ and average specific MLR at 19.2 g/sm^2^.

Besides lower values of mean HRR and average specific MLR, PMMA/SiO_2_@UiO-66 also shows a lower MAHRE than both PMMA/SiO_2_ and PMMA/UiO-66, indicating it has the best effect on reducing burning intensity, slowing down mass loss, and controlling fire spread. This effect is mainly ascribed to the protective layer formed from the thermal decomposition of additives. As shown in Figure 6d, after the cone calorimeter tests, a white layer is also formed from the burning of PMMA/SiO_2_@UiO-66, which, however, is thicker than the layer formed from the burning of PMMA/UiO-66 and more compact than the layer formed from the burning of PMMA/SiO_2_.

Given their very close values of CO yield, CO_2_ yield, EHC, and fire load, the dominant flame-retardant mechanism of UiO-66 and SiO_2_@UiO-66 for PMMA is related to physical actions in the condensed phase. The addition of UiO-66 and SiO_2_@UiO-66 will not lead to a significant change in the main flaming combustion reactions in the gaseous phase and will also not change the thermal decomposition mechanisms of PMMA significantly and contribute to promoting char formation in the condensed phase so that both the heat of combustion and combustion products will not be significantly changed. For the combustion residues left after the cone calorimeter tests, their PXRD patterns were analyzed to identify the chemical component. For PMMA/SiO_2_, its combustion residues are mainly composed of amorphous silica. Except for a broad peak centered at 2θ = 23°, there are no other characteristic peaks observed from its PXRD pattern shown in Figure 6e. The PXRD pattern of the combustion residue of PMMA/UiO-66 shows the characteristic peaks of ZrO_2_ [33]. The PXRD pattern of the combustion residue of PMMA/SiO_2_@UiO-66 shows the characteristic peaks of ZrO_2_, and the broad hump associated with the amorphous SiO_2_ can also be observed there.

### 3.4. Effect of UiO-66 on Thermal Decomposition Kinetics of PMMA

**Figure 7:**
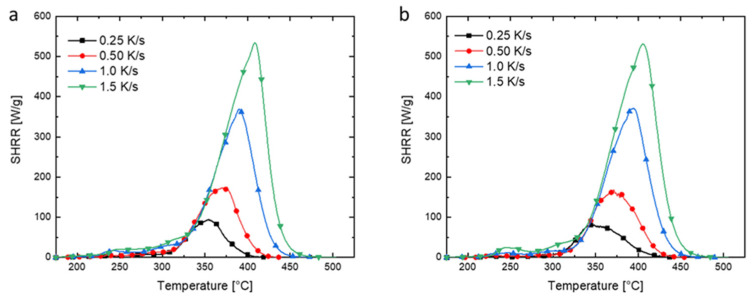
SHRR curves of neat PMMA (**a**) and PMMA/UiO-66 (**b**) at different heating rates under MCC tests.

The kinetic model of the material pyrolysis is the essential component of any comprehensive model for flaming ignition, burning, and flame spread over solid combustibles. Recently, MCC has been useful to study the kinetic models for the pyrolysis of flammable materials. In this study, PMMA and PMMA/UiO-66 were tested at four different heating rates: 0.25, 0.5, 1, and 1.5 K/s. Their MCC test results are shown in Figure 7. The specific heat release rate (SHRR) curve with respect to test temperature depends on the heating rate. For both PMMA and PMMA/UiO-66, with the increase in heating rate, both the peak specific heat release rate and the temperature at the peak specific heat release rate increase, due to the phenomenon of pyrolysis hysteresis [34].

At higher heating rates, several competing reactions occur simultaneously, and different reactions will overlap in the temperature range. Thus, given the thermal decomposition mechanisms of PMMA discussed earlier, the use of the single-step global reaction model is appropriate for determining the kinetic parameters of PMMA and PMMA/UiO-66, in which it is assumed that the thermal decomposition of the original sample only produces solid residual and combustible gas volatiles [35]. Thus, the reaction rate can be defined as Equation (1):

(1) $$\dot{r}=Af(\alpha )\exp(-\frac{E_{a}}{RT})$$

where $\dot{r}$ is reaction rate (1/s), *A* is the pre-exponential factor (1/s), *α* is the degree of conversion, *E_a_* is the activation energy (J/mol), *R* is the universal gas constant (8.314 J/mol·K), and *T* is the reaction temperature (K).

When processing MCC data, it is convenient to define the heat-release-based global conversion as Equation (2), and heat-release-based reaction rate is determined as Equation (3).

(2) $$\alpha_{q}=\frac{\int_{T_{0}}^{T}\dot{q}(T)dT}{\int_{T_{0}}^{\infty }\dot{q}(T)dT}$$

(3) $${\dot{r}}_{q}=\frac{d\alpha_{q}}{dt}$$

where *α_q_* is the heat-released-based global conversion, $\dot{q}$ is specific heat release rate (W/g), ${\dot{r}}_{q}$ is heat-release-based reaction rate (1/s), and *t* is time (s).

Based on the method proposed by Snegirev, the heat-release-based global conversion and reaction rate have proven to be suitable to derive the pyrolysis kinetic model for PMMA, and its MCC measurements can be replicated with good accuracy [36]. Then, because of its capability of obtaining reliable pyrolysis kinetic parameters without involving a kinetic model, the model-free Friedman method was applied to determine the kinetic parameters of thermal decomposition of PMMA and PMMA/UiO-66 using MCC results, in which the logarithmic form of Equation (1) is used as Equation (4).

(4) $$\ln r_{i}=-{\left(\frac{E_{a}}{RT}\right)}_{i}+\ln{\left(Af(\alpha )\right)}_{i}$$

where the subscript *i* corresponds to a particular conversion, *a_i_*. At a certain degree of conversion, for a single heating rate, Equation (4) yields a single point into the plot of ln *r_i_* versus 1/*T*. With different heating rates, *E_a_* can be derived from the slope of the plot of ln *r_i_* versus 1/*T* for each degree of conversion, α, regardless of the model. Based on the value of *E_a_*, how the addition of UiO-66 affects the thermal stability of PMMA can also be evaluated.

**Figure 8:**
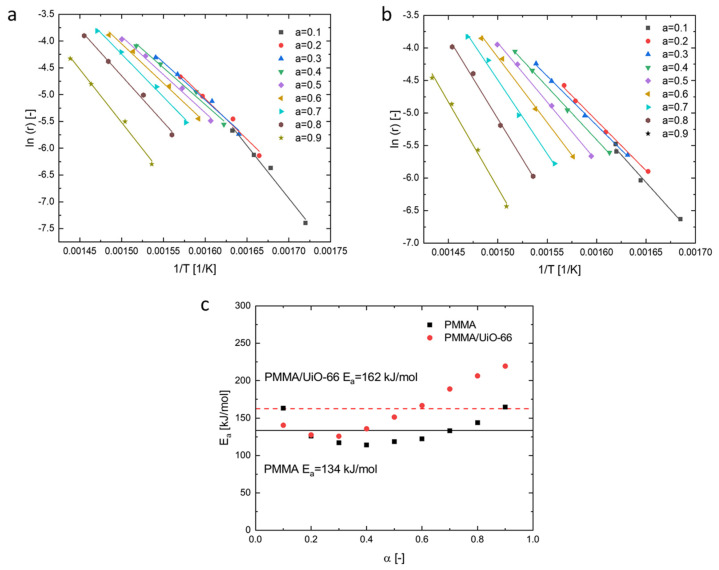
ln (*r_i_*) versus 1/*T* plots of neat PMMA (**a**) and PMMA/UiO-66 (**b**) at different degrees of conversion; (**c**) the dependencies of the apparent activation energy on the degree of conversion.

Based on the kinetic model described above, for PMMA and PMMA/UiO-66, the values of the apparent activation energy were calculated at different degrees of conversion and their dependencies on the degree of conversion are shown in Figure 8 with the conversions ranging from 0.1 to 0.9. Their average values of the activation energy are also shown there. The average apparent activation energy of PMMA is around 134 kJ/mol, which is in good agreement with the apparent activation energy determined for PMMA prepared by free radical initiators [37]. As a comparison, the average apparent activation energy of PMMA/UiO-66 is around 162 kJ/mol, which is higher than that of neat PMMA. The activation energy is the energy barrier that must be overcome to break chemical bonds and initiate decomposition processes [38]. Therefore, the initiation of thermal decomposition of PMMA/UiO-66 requires a larger amount of energy than neat PMMA, and thus it has better thermal stability. With the thermal decomposition reactions proceeding, the values of *E_a_* of PMMA/UiO-66 become continuously higher than those of neat PMMA. This is mainly due to the presence of UiO-66 in the PMMA matrix. With the melting of PMMA, UiO-66 migrates to the polymer surface, forms an effective diffusion barrier, and thus hinders the diffusion of formed gases from the thermal decomposition of PMMA. These results suppose that the shielding effect of UiO-66 could increase the activation energy of the polymer degradation [39].

## 4. Conclusions

In this study, UiO-66 and its composite with SiO_2_ were synthesized and well characterized first. Then, they were added into PMMA to form polymer composites via in situ polymerization. Based on the results from cone calorimeter tests, both UiO-66 and SiO_2_@UiO-66 show flame-retardant effects on PMMA, which is even better than nanosilica, a well-accepted, environmentally friendly flame-retardant filler for PMMA. With only 1.5 wt% of mass loading, it was found UiO-66 can reduce the maximum burning intensity and average mass loss rate of PMMA by 14.3% and 12.4%, respectively. Due to the synergistic effect between SiO_2_ and UiO-66 on forming a more compact protective layer in the condensed phase during the burning of PMMA, SiO_2_@UiO-66 can reduce the maximum burning intensity and average mass loss rate of PMMA by 22.0% and 14.7%, respectively. Based on the analysis of combustion gas emission and heat of combustion, the dominant flame-retardant mechanism of UiO-66 and SiO_2_@UiO-66 for PMMA is related to physical actions in the condensed phase. Because of its well-studied thermal decomposition mechanisms, PMMA was further used as the model polymer matrix to evaluate how the addition of MOF may affect its decomposition process and kinetics. Based on the results obtained from the microscale combustion calorimeter (MCC) at different heating rates, a heat-release-based kinetic model was used to determine the values of the apparent activation energy of neat PMMA and PMMA/UiO-66 composite at different degrees of conversion. The calculated apparent activation energy of neat PMMA shows good agreement with the previous research. It was found the average apparent activation energy of PMMA/UiO-66 (162 kJ/mol) is higher than that of neat PMMA (134 kJ/mol), indicating PMMA/UiO-66 has better thermal stability. This heat-release-based pyrolysis kinetic model can also be applied to other MOF-based polymer composites, which is useful for the modeling of their flaming ignition, burning, and flame spread.

## Figures and Tables

**Table 1:** TGA data of neat PMMA, PMMA/UiO-66, PMMA/SiO_2_, and PMMA/SiO_2_@UiO-66 in nitrogen atmosphere.

Samples	T_onset_ (°C)	T_max_ (°C)	Mass Residue at T_max_ (wt%)	Peak Mass Loss Rate (wt%/°C)	Residue at 600 °C (wt%)
PMMA	293	394	26.9	1.57	0.6
PMMA/UiO-66	297	395	25.9	1.54	0.8
PMMA/SiO_2_	290	383	33.6	1.20	2.3
PMMA/SiO_2_@UiO-66	288	397	24.3	1.39	1.6

**Table 2:** Cone calorimeter test data of neat PMMA, PMMA/UiO-66, PMMA/SiO_2_, and PMMA/SiO_2_@UiO-66.

Sample	Time to Ignition (s)	pHRR (kW/m^2^)	Time to pHRR (s)	Mean HRR (kW/m^2^)	Average Specific MLR (g/sm^2^)	Mean CO Yield (kg/kg)	Mean CO_2_ Yield (kg/kg)	EHC (MJ/kg)	MARHE (kW/m^2^)	Fuel Load (MJ/kg)
PMMA	19	832	195	307	22.5	0.0146	1.87	23.89	680.4	23.81
PMMA/UiO-66	20	713	275	283	19.7	0.0136	1.83	23.87	537.3	23.55
PMMA/SiO_2_	14	758	220	303	24.0	0.0127	2.18	23.53	551.5	23.36
PMMA/SiO_2_@UiO-66	25	649	228	277	19.2	0.0139	1.79	23.51	504.7	23.35

## Data Availability

Not applicable.

## References

[1] Zhu Q.-L., Xu Q. (2014). Metal-organic framework composites. Chem. Soc. Rev..

[2] Kuppler R.J., Timmons D.J., Fang Q.-R., Li J.-R., Makal T.A., Young M.D., Yuan D., Zhao D., Zhuang W., Zhou H.-C. (2009). Potential applications of metal-organic frameworks. Coord. Chem. Rev..

[3] Nabipour H., Wang X., Song L., Hu Y. (2020). Metal-organic frameworks for flame retardant polymers application: A critical review. Compos. Part A Appl. Sci. Manuf..

[4] Hou Y., Xu Z., Chu F., Gui Z., Song L., Hu Y., Hu W. (2021). A review on metal-organic hybrids as flame retardants for enhancing fire safety of polymer composites. Compos. B Eng..

[5] Pan Y.-T., Zhang Z., Yang R. (2020). The rise of MOFs and their derivatives for flame retardant polymeric materials: A critical review. Compos. B Eng..

[6] Korobeinichev O.P., Paletsky A.A., Gonchikzhapov M.B., Glaznev R.K., Gerasimov I.E., Naganovsky Y.K., Shundrina I.K., Snegirev A.Y., Vinu R. (2019). Kinetics of thermal decomposition of PMMA at different heating rates and in a wide temperature range. Thermochim. Acta.

[7] Bhargava A., van Hees P., Andersson B. (2016). Pyrolysis modeling of PVC and PMMA using a distributed reactivity model. Polym. Degrad. Stab..

[8] Luche J., Rogaume T., Richard F., Guillaume E. (2011). Characterization of thermal properties and analysis of combustion behavior of PMMA in a cone calorimeter. Fire Saf. J..

[9] Bai Y., Dou Y., Xie L.-H., Rutledge W., Li J.-R., Zhou H.-C. (2016). Zr-based metal-organic frameworks: Design, synthesis, structure, and applications. Chem. Soc. Rev..

[10] Yang D., Hu Y., Song L., Nie S., He S., Cai Y. (2008). Catalyzing carbonization function of α-ZrP based intumescent fire retardant polypropylene nanocomposites. Polym. Degrad. Stab..

[11] Ma M., Lu L., Li H., Xiong Y., Dong F. (2019). Functional metal organic framework/SiO_2_ nanocomposites: From versatile synthesis to advanced applications. Polymers.

[12] Zhang W., Yan Z., Gao J., Tong P., Liu W., Zhang L. (2015). Metal-organic framework UiO-66 modified magnetite@silica core-shell magnetic microspheres for magnetic solid-phase extraction of domoic acid from shellfish samples. J. Chromatogr. A.

[13] Shen R., Hatanaka L.C., Ahmed L., Agnew R.J., Mannan M.S., Wang Q. (2017). Cone calorimeter analysis of flame retardant poly (methyl methacrylate)-silica nanocomposites. J. Therm. Anal. Calorim..

[14] Rantuch P., Martinka J., Ház A. (2021). The evaluation of torrefied wood using a cone calorimeter. Polymers.

[15] Lazar S., Shen R., Quan Y., Palen B., Wang Q., Ellison C.J., Grunlan J.C. (2021). Mixed solvent synthesis of polydopamine nanospheres for sustainable multilayer flame retardant nanocoating. Polym. Chem..

[16] Guo J.Z., Song K., Liu C. (2018). Polymer-Based Multifunctional Nanocomposites and Their Applications.

[17] Ng Y.H., Zope I.S., Dasari A., Tan K.H. (2020). Correlating the performance of a fire-retardant coating across different scales of testing. Polymers.

[18] Xu Q., Jin C., Majlingova A., Restas A. (2018). Discuss the heat release capacity of polymer derived from microscale combustion calorimeter. J. Therm. Anal. Calorim..

[19] Gao B., Huang M., Zhang Z., Yang Q., Su B., Yang Y., Ren Q., Bao Z. (2019). Hybridization of metal–organic framework and monodisperse spherical silica for chromatographic separation of xylene isomers. Chin. J. Chem. Eng..

[20] Yang F., Xie S., Wang G., Yu C.W., Liu H., Liu Y. (2020). Investigation of a modified metal-organic framework UiO-66 with nanoscale zero-valent iron for removal of uranium (VI) from aqueous solution. Environ. Sci. Pollut. Res. Int..

[21] Yang P., Liu Q., Liu J., Zhang H., Li Z., Li R., Liu L., Wang J. (2017). Interfacial growth of a metal–organic framework (UiO-66) on functionalized graphene oxide (GO) as a suitable seawater adsorbent for extraction of uranium(vi). J. Mater. Chem. A.

[22] Guo W., Nie S., Kalali E.N., Wang X., Wang W., Cai W., Song L., Hu Y. (2019). Construction of SiO_2_@UiO-66 core–shell microarchitectures through covalent linkage as flame retardant and smoke suppressant for epoxy resins. Compos. B Eng..

[23] Fu Y.-Y., Yang C.-X., Yan X.-P. (2013). Fabrication of ZIF-8@SiO_2_ core-shell microspheres as the stationary phase for high-performance liquid chromatography. Chemistry.

[24] Sun T., Zhuo Q., Liu X., Sun Z., Wu Z., Fan H. (2014). Hydrophobic silica aerogel reinforced with carbon nanotube for oils removal. J. Porous Mater..

[25] Zhang X., Han Q., Ding M. (2015). One-pot synthesis of UiO-66@SiO_2_ shell–core microspheres as stationary phase for high performance liquid chromatography. RSC Adv..

[26] Yang Q., Zhang H.-Y., Wang L., Zhang Y., Zhao J. (2018). Ru/UiO-66 catalyst for the reduction of nitroarenes and tandem reaction of alcohol oxidation/Knoevenagel condensation. ACS Omega.

[27] Shi X., Dai X., Cao Y., Li J., Huo C., Wang X. (2017). Degradable poly(lactic acid)/metal–organic framework nanocomposites exhibiting good mechanical, flame retardant, and dielectric properties for the fabrication of disposable electronics. Ind. Eng. Chem. Res..

[28] Ferriol M., Gentilhomme A., Cochez M., Oget N., Mieloszynski J.L. (2003). Thermal degradation of poly(methyl methacrylate) (PMMA): Modelling of DTG and TG curves. Polym. Degrad. Stab..

[29] Li A., Xu W., Chen R., Liu Y., Li W. (2018). Fabrication of zeolitic imidazolate frameworks on layered double hydroxide nanosheets to improve the fire safety of epoxy resin. Compos. Part A Appl. Sci. Manuf..

[30] Hu Y.-H., Chen C.-Y., Wang C.-C. (2004). Viscoelastic properties and thermal degradation kinetics of silica/PMMA nanocomposites. Polym. Degrad. Stab..

[31] Schartel B., Hull T.R. (2007). Development of fire-retarded materials—Interpretation of cone calorimeter data. Fire Mater..

[32] Ahmed L., Zhang B., Shen R., Agnew R.J., Park H., Cheng Z., Mannan M.S., Wang Q. (2018). Fire reaction properties of polystyrene-based nanocomposites using nanosilica and nanoclay as additives in cone calorimeter test. J. Therm. Anal. Calorim..

[33] Basahel S.N., Ali T.T., Mokhtar M., Narasimharao K. (2015). Influence of crystal structure of nanosized ZrO_2_ on photocatalytic degradation of methyl orange. Nanoscale Res. Lett..

[34] Xu Y., Yang Y., Shen R., Parker T., Zhang Y., Wang Z., Wang Q. (2019). Thermal behavior and kinetics study of carbon/epoxy resin composites. Polym. Compos..

[35] Snegirev A.Y., Talalov V.A., Stepanov V.V., Korobeinichev O.P., Gerasimov I.E., Shmakov A.G. (2017). Autocatalysis in thermal decomposition of polymers. Polym. Degrad. Stab..

[36] Snegirev A.Y. (2014). Generalized approach to model pyrolysis of flammable materials. Thermochim. Acta.

[37] Holland B.J., Hay J.N. (2002). The effect of polymerisation conditions on the kinetics and mechanisms of thermal degradation of PMMA. Polym. Degrad. Stab..

[38] Lyon R.E., Safronava N. (2013). A comparison of direct methods to determine n-th order kinetic parameters of solid thermal decomposition for use in fire models. J. Therm. Anal. Calorim..

[39] Benhacine F., Yahiaoui F., Hadj-Hamou A.S. (2014). Thermal stability and kinetic study of isotactic polypropylene/Algerian bentonite nanocomposites prepared via melt blending. J. Polym..

